# Working Towards a Treat-to-Target Protocol in Juvenile Proliferative Lupus Nephritis – A Survey of Pediatric Rheumatologists and Nephrologists in Germany and Austria

**DOI:** 10.3389/fped.2022.851998

**Published:** 2022-04-22

**Authors:** Kristina Vollbach, Catharina Schuetz, Christian M. Hedrich, Fabian Speth, Kirsten Mönkemöller, Jürgen Brunner, Ulrich Neudorf, Christoph Rietschel, Anton Hospach, Tilmann Kallinich, Claas Hinze, Norbert Wagner, Burkhard Tönshoff, Lutz T. Weber, Kay Latta, Julia Thumfart, Martin Bald, Dagobert Wiemann, Hildegard Zappel, Klaus Tenbrock, Dieter Haffner

**Affiliations:** ^1^Department of Pediatrics, RWTH Aachen University Hospital, Aachen, Germany; ^2^Department of Pediatrics, Medizinische Fakultät Carl Gustav Carus, Technische Universität Dresden, Dresden, Germany; ^3^Department of Pediatric Rheumatology, Alder Hey Children’s NHS Foundation Trust Hospital, Liverpool, United Kingdom; ^4^Department of Women’s and Children’s Health, Institute of Life Course and Medical Sciences, University of Liverpool, Liverpool, United Kingdom; ^5^Universitätsmedizin Hamburg, Kinder- und Jugendklinik, Hamburg, Germany; ^6^Department of Pediatrics, Kinderkrankenhaus Amsterdamer Strasse, Cologne, Germany; ^7^Department of Pediatrics, Pediatric Rheumatology, Medizinische Universität Innsbruck, Innsbruck, and Danube Private University, Krems an der Donau, Austria; ^8^Clinic for Pediatrics III, University Hospital Essen, Essen, Germany; ^9^Department of Pediatric Rheumatology, Clementine Kinderhospital, Frankfurt, Germany; ^10^Center for Pediatric Rheumatology, Olgahospital, Stuttgart, Germany; ^11^German Rheumatism Research Center, Leibniz Institute, Berlin, and Charité Universitätsmedizin Berlin, Pediatric Pneumology, Immunology and Critical Care Medicine and SPZ (Center for Chronically Sick Children), Berlin, Germany; ^12^Department of Pediatric Rheumatology and Immunology, University Hospital, Münster, Germany; ^13^Department of Pediatrics I, University Children’s Hospital, Heidelberg, Germany; ^14^Division of Pediatric Nephrology, Children’s and Adolescents’ Hospital, University Hospital of Cologne, Faculty of Medicine, University of Cologne, Cologne, Germany; ^15^Clementine Kinderhospital Frankfurt, Frankfurt, Germany; ^16^Department of Pediatric Gastroenterology, Nephrology and Metabolic Diseases, Charité Universitätsmedizin Berlin, Berlin, Germany; ^17^Division of Pediatric Nephrology, Olgahospital, Klinikum Stuttgart, Stuttgart, Germany; ^18^Division of Pediatric Diabetology/Endocrinology, University Hospital Magdeburg, Magdeburg, Germany; ^19^Clinic of Pediatrics and Adolescent Medicine, University Medical Center Göttingen, Göttingen, Germany; ^20^Department of Pediatric Kidney, Liver and Metabolic Diseases, Hannover Medical School, Hanover, Germany

**Keywords:** SLE, nephritis, T2T, mycophenolate mofetil, cyclophosphamide, corticosteroid, kidney biopsy

## Abstract

**Background:**

To describe treatment practices for juvenile proliferative lupus nephritis (LN) class III and IV of pediatric rheumatologists and nephrologists in Germany and Austria in preparation for a treat-to-target treatment protocol in LN.

**Methods:**

Survey study by members of the Society for Pediatric and Adolescent Rheumatology (GKJR) and the German Society for Pediatric Nephrology (GPN) on diagnostics and (concomitant) therapy of LN.

**Results:**

Fifty-eight physicians completed the survey. Overall, there was a considerable heterogeneity regarding the suggested diagnostics and management of juvenile proliferative LN. Increased urinary protein excretion, either assessed by 24 h urine collection or spot urine (protein-creatinine ratio), and reduced estimated glomerular filtration rate were specified as important parameters for indication of kidney biopsy to diagnose proliferative LN and monitoring of therapy. Corticosteroids were generally proposed for induction and maintenance therapy, most often in conjunction with either mycophenolate mofetil (MMF) or cyclophosphamide (CP) as steroid-sparing immunosuppressants. MMF was clearly preferred over CP for induction therapy of LN class III, whereas CP and MMF were equally proposed for LN class IV. MMF was most often recommended for maintenance therapy in conjunction with oral corticosteroids and continued for at least 3 years and 1 year, respectively, after remission. Hydroxychloroquine was widely accepted as a concomitant measure followed by renin-angiotensin system inhibitors in cases of arterial hypertension and/or proteinuria.

**Conclusion:**

The majority of pediatric rheumatologists and nephrologists in Germany and Austria propose the use of corticosteroids, most often in combination with either MMF or CP, for treatment of proliferative LN in children. The considerable heterogeneity of responses supports the need for a treat-to-target protocol for juvenile proliferative LN between pediatric rheumatologists and nephrologists.

## Introduction

Lupus nephritis (LN) is a substantial cause of morbidity and mortality among patients with systemic lupus erythematosus (SLE). Within 10 years of an initial SLE diagnosis, 5–20% of patients with LN develop end-stage kidney disease (1). In up to 20% of all SLE patients, the onset of the disease occurs in childhood or adolescence (2). By contrast to adults, 50 to 60% of patients with juvenile onset SLE will develop lupus nephritis (3–6).

In German registries [National Pediatric Rheumatologic Database and German Lupus Nephritis Registry of the German Society for Pediatric Nephrology (GPN)], approximately 20 patients with LN per year are newly documented (with the possibility of underrepresentation due to the level of awareness of the registries) (7). The small number of cases distributed over several centers hampers a standardized procedure for this difficult-to-treat disease.

To improve long-term outcomes in children and adolescents with rheumatic diseases, the definition and evaluation of therapeutic strategies (treat-to-target, T2T) is an important tool (8). To develop these tools, the PRO-KIND initiative (Projekte zur Klassifikation, Überwachung und Therapie in der Kinderrheumatologie/Projects on classification, monitoring and therapy in pediatric rheumatology) within the Commission of the Society for Pediatric and Adolescent Rheumatology (Gesellschaft für Kinder- und Jugendrheumatologie, GKJR) was founded in 2015. Their task is to develop T2T protocols for the most prevalent pediatric rheumatic diseases in Germany, some of which have been published (9–15). In 2019, the initiative received funding from the Joint Federal Committee (Gemeinsamer Bundesausschuss, GBA) to evaluate the practicability and effectiveness of these protocols. To achieve this aim, 500 patients with new-onset rheumatic diseases will be recruited in a register study and treatment of these patients will be prospectively followed for 12 months. The register is currently recruiting patients until September 2022.

For SLE and specifically for LN, there is currently no T2T therapy protocol, while consensus treatment plans (CTPs) of the Childhood Arthritis and Rheumatology Research Alliance (CARRA) and the Single Hub and Access point for pediatric Rheumatology in Europe (SHARE) initiative’s recommendations are available (16, 17). Therefore, the development of an agreed consensus treatment protocol between pediatric rheumatologists and nephrologists is a key goal. To develop such a protocol, the PRO-KIND SLE working group evolved and conducted a survey together with the SLE working group of the GPN. This survey addressed current diagnosis and treatment of patients with proliferative LN in Germany and Austria on the basis of different case vignettes. The survey was distributed *via* the mailing lists of GKJR and GPN, and 58 German-speaking pediatric rheumatologists and nephrologists completed this survey and the data is presented here.

## Materials and Methods

### Survey

A survey (Supplementary Appendix) was developed by the PRO-KIND SLE working group and representatives of the GPN. The survey consisted of 25 closed and open-ended questions. Questions (a combination of Likert scale, multiple choice, and open comments) included:

– The respondent’s field of activity and type of workplace, as well as their age group
– Whether the respondents are currently treating patients with SLE or how many they have treated in their career so far
– The description of patients with LN WHO class III or IV and further detailed questions on

∘ Diagnostics,
∘ Indication for kidney biopsy in SLE patients,
∘ Activity assessment of SLE,
∘ Therapy for proliferative LN class III or IV,
∘ Definition of response to therapy in LN and,
∘ Concomitant therapies and preventive measures in SLE or LN.

In August 2016, the survey link was mailed to pediatric rheumatologists and nephrologists *via* the mailing lists of GKJR and GPN. Responses were collected *via* SurveyMonkey^®^; a follow-up message was sent out once to encourage survey completion after a few weeks. Data collection was closed in January 2017.

### Analysis

Percentages and mean values were determined from the Likert scale and multiple-choice question responses. Rating averages were calculated by adding up the score results of the Likert scale and dividing it by the number of respondents. Open-ended comments were analyzed.

### Case Vignette

A 15-year-old previously healthy girl with a suspected diagnosis of SLE meeting 7 of 11 American College of Rheumatology (ACR) classification criteria (18) (malar rash, photosensitivity, oral ulcers, renal disorder, hematologic disorder, positive ANA titer, and immunologic disorder) was presented in a case vignette once with LN class III and once with LN class IV. Based on this case vignette, questions regarding further diagnostic and therapeutic procedures were queried in the survey (see Supplementary Appendix for full survey).

## Results

### Respondent Demographics

**FIGURE 1:**
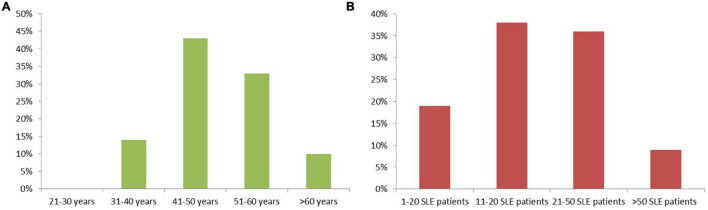
**(A)** Age distribution of survey respondents, and **(B)** numbers of SLE patients treated by each survey respondent.

Of all pediatric rheumatologists (*n* = 129) and nephrologists (*n* = 315) contacted, a total of 58 responded and 42 fully completed the survey, resulting in a response rate of 13 and 9.5%, respectively. Most respondents (*n* = 25) belong to the age group of 41–50 years, the others belong to the age groups 51–60 years (*n* = 19), 31–40 years (*n* = 8) and >60 years (*n* = 6), respectively (Figure 1A).

The participants were mostly pediatric rheumatologists (60%), followed by pediatric nephrologists (35%) and general pediatricians (5%). More than half (54%) work at a university hospital, 44% at a non-university hospital, and one participant in a general pediatric practice.

Of the participants 38% have cared for 11–20 SLE patients in their career to date, while 36% have cared for 21–50 SLE patients, 19% have treated 1–10 and 7% have treated more than 50 SLE patients (Figure 1B).

### Diagnostics

The vast majority of survey respondents (91%) would involve a pediatric nephrologist in the initial diagnosis and treatment planning of a patient with LN; other disciplines involved by the majority are ophthalmologists and cardiologists (see Table 1). 55% of respondents would suggest consulting a pediatric rheumatologist, which suggests that, depending on the presentation of the patient, pediatric rheumatologists are less frequently involved in the initial diagnosis and treatment planning.

**TABLE 1:** Consultants involved in the diagnosis and treatment planning of an SLE patient with suspected LN.

Discipline	Total number of respondents (%)
Pediatric nephrologist	38 (91)
Ophthalmologist (with fundus)	29 (69)
Pediatric cardiologist	24 (57)
Pediatric rheumatologist	23 (55)
Pediatric pulmonologist	11 (26)
Hematologist/Haemostaseologist	8 (19)
Neuropediatrician	7 (16)
Dermatologist	6 (14)
Psychologist/Psychiatrist	4 (9)
Gynecologist	2 (5)
Pediatric endocrinologist	1 (2)

When assessing the extent of LN, protein excretion in 24 h collection urine (>300 mg/m^2^ and 24 h or ≥0.5 g per 24 h) was rated as most essential, above the protein-creatinine ratio in spot urine (>0.2 g/g or >20 mg/mmol). However, several other parameters, e.g., dip-stick protein, and serum creatinine were also considered helpful (see Table 2).

**TABLE 2:** Laboratory parameters in terms of their importance in assessing the extent of LN (answers by Likert scale: 1 = very important, 2 = somewhat important, 3 = not very important, 4 = not important at all; with rating average of respondents).

	1 = very important; total number of respondents	2 = somewhat important; total number of respondents	3 = not very important; total number of respondents	4 = not important at all; total number of respondents	Rating average
24 h collection urine: protein excretion > 300 mg/m^2^ per 24 h or ≥0.5 g/1.73 m^2^ per 24 h	36	4	2	0	1.19
Spot urine collection: protein-creatinine ratio > 0.2 g/g (>20 mg/mmol) creatinine	29	13	0	0	1.31
Serum creatinine	26	13	2	0	1.41
Urine dip-stick: protein > twofold positive	19	19	4	0	1.64
Schwartz formula: estimated glomerular filtration rate (eGFR)	18	16	6	0	1.70
Cystatin C: estimated GFR (eGFR)	15	19	6	2	1.88
Creatinine clearance/BSA using 24 h collection urine	10	21	7	2	2.03

### Kidney Biopsy for Diagnosis of Lupus Nephritis

According to the majority of the respondents (98%), the decision to perform a kidney biopsy is made on the basis of relevant pathological urine and kidney findings. A pathologic urine finding requiring kidney biopsy was agreed upon by 100% (*n* = 42) of respondents for nephrotic-range proteinuria [>1 g/m^2^ per 24 h or protein-creatinine ratio > 2 g/g creatinine (>200 mg/mmol)], 98% (*n* = 41) for rapidly progressive proteinuria, 62% (*n* = 26) for pathologic urine status (e.g., >5 erythrocytes/high power field and/or detection of RBC casts) with mild-moderate proteinuria (≤1 g/m^2^ per 24 h or protein-creatinine ratio 0.2–2 g/g creatinine or 20–200 mg/mmol), 48% for mild-moderate proteinuria with normal urine status, and 24% for pathologic urine status without mild-moderate proteinuria. Pediatric nephrologists tended to propose more frequently isolated mild-moderate proteinuria as an indicator for kidney biopsy as compared to rheumatologists (60 vs. 45%). Parameters also considered important for the indication of a kidney biopsy are listed in Table 3, including reduced eGFR < 60 ml/min per 1.73 m^2^ and an increase in serum creatinine. By contrast, highly elevated double-stranded DNA antibodies alone and patients’ ethnicity were considered not very important.

**TABLE 3:** Other relevant parameters for the indication of a kidney biopsy (answers by Likert scale: 1 = very important, 2 = somewhat important, 3 = not very important, 4 = not important at all; with rating average of respondents).

	1 = very important; total number of respondents	2 = somewhat important; total number of respondents	3 = not very important; total number of respondents	4 = not important at all; total number of respondents	Rating average
eGFR < 60 ml/min per 1.73 m^2^	37	3	0	1	1.15
Elevated serum creatinine levels	33	6	0	1	1.23
eGFR < 90 ml/min per 1.73 m^2^	24	15	1	1	1.49
Elevated blood pressure (>95th percentile)	20	13	6	2	1.76
Combination of high auto-antibodies (dsDNA and/or nucleosomes) plus decreased complement levels (C3 and/or C4)	15	11	12	2	2.03
Strongly decreased C3	11	14	12	2	2.13
Strongly decreased C4	10	13	13	2	2.18
Patient ethnicity (African–American, Hispanic, Asian)	7	13	14	5	2.44
Strongly increased anti-dsDNA	6	11	11	9	2.62

### Activity Assessment of Systemic Lupus Erythematosus

To monitor SLE activity, more than 90% of respondents suggest the use of the Systemic Lupus Erythematosus Disease Activity Index 2000 (SLEDAI-2K) as a validated tool; further, Physician Global Assessment (76%), Parent/Patient Global Assessment (64%), and Childhood Health Assessment Questionnaire (C-HAQ) (52%) were considered relevant. Other tools such as Simple Measure of Impact of Lupus Erythematosus in Youngsters (SMILEY), which is not available in German, and European Consensus Lupus Activity Measurement (ECLAM) (10% each) and British Isles Lupus Assessment Group (BILAG) index (5%) were considered less relevant.

### Therapy for Systemic Lupus Erythematosus With Proliferative Lupus Nephritis Class III

As the answers on therapy schemes were a combination of Likert scale, multiple choice, and open comments, the different dosing regimens are displayed in the Supplementary Appendix. Only significant differences are described, otherwise no clear preference for the proposed dosing regimens were seen.

The hypothetical patient with LN outlined in the case vignette, presented with proteinuria 500 mg/m^2^ and protein-creatinine ratio 0.8 g/g creatinine (90 mg/mmol), erythrocyte cylinder Erys: 10/high power field, eGFR: 110 ml/min per 1.73 m^2^ and blood pressure at the 75th percentile.

For **induction** therapy for the SLE patient with LN class III, as outlined in the case vignette, 54% (*n* = 22) of respondents opted for the combination of intravenous and oral corticosteroid therapy (Table 4), and similar numbers suggest mainly intravenous (*n* = 10) or mainly oral (*n* = 9) therapy.

**TABLE 4:** Suggested corticosteroid induction therapies for proliferative lupus nephritis class III or IV (adapted from Refs. 17, 63).

	Prednisolone/methylprednisolone (PDN/MP) therapy in the first 6 months
Mainly intravenous (i.v.)	MP i.v. 15–30 mg/kg (max 1 g) or 300–500 mg/m^2^ for 3 days, then i.v. MP pulse therapy initially 1x/week, then 1x/month+ start PDN *per os* (p.o.) 0.5 mg/kg → reduction → target PDN 6–10 mg/m^2^/48 h or 0.2 mg/kg (up to max 10 mg/d) p.o.
Mainly p.o.	MP once i.v. 15–30 mg/kg (max 1 g) or 300–500 mg/m^2^ for 3 days+ start PDN p.o. 2 mg/kg or 60 mg/m^2^ for 6 weeks → reduction → target PDN 6–10 mg/m^2^/48 h or 0.5 mg/kg (max 20 mg/d)
Combined i.v. + p.o.	MP i.v. 15–30 mg/kg (max 1 g) or 300–500 mg/m^2^ for 3 days, further optional i.v. MP pulse therapy (max 1x/month)+ p.o. PDN start 1(–1.5)mg/kg → reduction by 10% every (1–)2 weeks → target PDN 6–10 mg/m^2^/48 h or 0.2 mg/kg (up to max. 15 mg/d)

For non-steroidal immunosuppressive therapy (NSI), 74% respondents (*n* = 31) suggest initiation of therapy with mycophenolate mofetil (MMF) at a dosage of 1000–1200 mg/m^2^ per day, with either a maximum of 3 g (*n* = 15) or 2 g per day (*n* = 16). The lower MMF dosage was more frequently proposed by pediatric rheumatologists compared to nephrologists (50 vs. 27%). Other suggested additional immunosuppressants (multiple answers possible, see Supplementary Appendix for dosing regimens) were cyclophosphamide (CP) [*n* = 7 (17%), with no clear preference for the preferred dosage], azathioprine (AZA), and rituximab (RTX) (*n* = 3 each). Another 3 respondents indicated that they would not use any other immunosuppression in addition to corticosteroid therapy. In the open-ended comments, hydroxychloroquine (HCQ) was mentioned as an immunomodulatory. This concomitant drug for SLE was not part of the selection, as it is not specifically for the treatment of LN. Primary therapy with cyclosporin A (CsA) was not suggested by any respondent.

For **maintenance** therapy after achieving disease inactivity and after at least 6 months, 39% (*n* = 16) of respondents opted to discontinue corticosteroid therapy upon complete remission of the nephritis (based on normalization of proteinuria and eGFR) and 61% (*n* = 25) decided to continue corticosteroid therapy for at least one more year. There was no clear consensus for PDN dosing in maintenance therapy, with half of the subgroup opting for 5–7.5 mg per day (or 0.15–0.2 mg/kg per day). BSA adjusted corticosteroid dosing was preferred by pediatric nephrologists, whereas rheumatologists preferred dosing according to body weight. For further immunosuppression in the context of maintenance therapy, most respondents (*n* = 24, 57%) suggested MMF (with a dosage of 1000–1200 mg/kg per day, max. 2 g per day). 8 respondents (20%) would prefer therapy with AZA, 3 (8%) with RTX (with no clear preference for the proposed dosing regimens), and 2 (5%) with CsA.

In case of non-response to induction therapy in LN class III, 5% of respondents (*n* = 2) suggest continuing the basic medication and only increase PDN p.o. or MP i.v. dose. By contrast, the remaining 95% of respondents (*n* = 39) would add an immunosuppressive drug, depending on the previous therapy, in addition to corticosteroids. In general (multiple answers possible), 16 respondents (32%) suggested CP, another 16 respondents (32%) suggested MMF (see Supplementary Appendix for suggested dosage regimens, no clear preference emerged among respondents), 11 (22%) suggested RTX, and 7 (14%) CsA. Two respondents proposed plasmapheresis and one immunoadsorption. In the open comments, therapy with ofatumumab (alone or in combination with MMF) was suggested twice.

### Therapy for Systemic Lupus Erythematosus With Proliferative Lupus Nephritis Class IV

The hypothetical patient with LN outlined in the case vignette presented with prognostically unfavorable risk factors: histology with LN WHO class IV and 50% crescent formation, proteinuria 1.5 g/m^2^ per day and urinary protein-creatinine ratio 2.1 g/g creatinine (237 mg/mmol), erythrocyte cylinder 10/high power field, eGFR 72 ml/min per 1.73 m^2^, blood pressure 97th percentile.

For **induction** therapy, 67% of respondents (*n* = 28) would opt for a combined i.v. and p.o. corticosteroid therapy and 33% (*n* = 14) for oral therapy only. For additional immunosuppression (multiple answers possible), a similar number of respondents suggested the use of CP (*n* = 23, 40%), including 3 in combination with other immunosuppressants and MMF (*n* = 24, 41%). Other immunosuppressants or therapies suggested were RTX (*n* = 8, 14%), CsA (*n* = 1, 2%), MTX (*n* = 1, 2%), and plasmapheresis (*n* = 1, 2%).

For **maintenance** therapy after achieving disease inactivity and after at least 6 months, 82% (*n* = 31) proposed continuation of corticosteroid therapy for at least one more year. Unlike in LN class III, most respondents (*n* = 16, 42%) suggested a dose of 5–7.5 mg per day (or 0.15–0.2 mg/kg/d), 18% (*n* = 7) of respondents opted to discontinue corticosteroid therapy following complete clinical remission of nephritis. Furthermore, most respondents suggest MMF (*n* = 34, 69%) for maintenance therapy (multiple answers possible) and others suggest RTX (*n* = 6, 12%), AZA (*n* = 6, 12%), CsA (*n* = 3, 6%), and CP (*n* = 1, 2%).

In case of non-response to induction therapy, most respondents (*n* = 37, 97%) opt for an extension of the basic therapy beyond the increase of the corticosteroid dose, depending on previous therapy. This most often included (multiple answers possible) RTX (*n* = 21), followed by CP (*n* = 17), MMF (*n* = 11), CsA (*n* = 6), AZA (*n* = 3), plasmapheresis (*n* = 6), and immunoadsorption (*n* = 3). In the open comments, therapy with ofatumumab (alone or in combination with MMF) was indicated twice, as well as therapy with intravenous immunoglobulin (IVIG) (*n* = 1) and tacrolimus (*n* = 1) and combinations of immunosuppressants, e.g., MPN + RTX + MMF + CsA or RTX + MMF.

### Definition of Response to Therapy in Lupus Nephritis

Parameters most frequently considered relevant for assessing a satisfactory response to LN therapy were either the urinary protein-creatinine ratio in a spot urine, or 24 h protein excretion and normalization of eGFR (>90 ml/min per 1.73 m^2^). However, normalization of urine sediment and normalization of serum complement C3 were also considered important decision tools (see Table 5).

**TABLE 5:** Suggested criteria in assessing remission in lupus nephritis (answers by Likert scale: 1 = very important, 2 = somewhat important, 3 = not very important, 4 = not important at all; with rating average of respondents).

	1 = very important; total number of respondents	2 = somewhat important; total number of respondents	3 = not very important; total number of respondents	4 = not important at all; total number of respondents	Rating average
Protein-creatinine ratio < 0.2 g/g (<20 mg/mmol) crea or protein excretion < 200 mg/24 h in 24 h urine collection	31	9	0	0	1.23
eGFR > 90 ml/min per 1.73 m^2^	23	14	0	0	1.38
Normalization of serum complement C3	16	21	4	0	1.71
Urine sediment (erythrocytes 5/high power field, no RBC casts detectable)	15	19	6	0	1.78
SLEDAI score < 2	3	23	10	1	2.24

As shown in Table 5, remission was defined by the following criteria: protein-creatinine ratio <0.2 g/g creatinine (<20 mg/mmol), eGFR > 90 ml/min per 1.73 m^2^, and SLEDAI score < 2. Time acceptable to achieve remission was selected by participants.

In case of LN class III, 71% (*n* = 29) of participants considered an interval of 8–12 weeks until remission, following induction therapy, as acceptable. Seven% of respondents considered an interval of 24 weeks as acceptable, while 5% would expect a therapeutic response after only 2 weeks, or 17% after 4 weeks, following induction therapy.

In cases of LN class IV, 49% (*n* = 20) of respondents considered a period of 8–12 weeks until reaching a therapeutic response (see Table 5) to be acceptable, whereas 5% would require this after only 2 weeks, or 34% after 4 weeks, following induction therapy, and 10% stated the acceptable interval until response to be 24 weeks and 2% even 52 weeks.

In case of a satisfactory treatment response, most respondents (64%, *n* = 27) suggest continuing immunosuppressive therapy for at least 3 years, whereas 10% (*n* = 4) suggest stopping treatment after 1 year and 26% (*n* = 11) suggest continuing for more than 3 years. Pediatric nephrologists proposed longer treatment durations.

Table 6 shows different scenarios for repeat kidney biopsies. Respondents saw different indications, but most would rather perform a repeat kidney biopsy in the event of a suspected recurrence of nephritis. Other reasons included persistence of proteinuria for over one year on maintenance therapy, partial response after 6–12 months, or after 3–4 months in case of non-response at the end of induction therapy.

**TABLE 6:** Indications for repeat kidney biopsy during follow-up of lupus nephritis (answers by Likert scale: 1 = fully agree, 2 = tend to agree, 3 = partly/partly, 4 = tend to disagree, 5 = do not agree; with rating average of respondents).

	1 = fully agree; total number of respondents	2 = tend to agree; total number of respondents	3 = partly/partly; total number of respondents	4 = tend to disagree; total number of respondents	5 = do not agree at all; total number of respondents	Rating average
In case of suspected recurrence of nephritis	16	16	10	0	0	1.86
In maintenance therapy if proteinuria persists > 1 year	13	15	7	4	0	2.05
Persistent eGFR < 90 ml/min per 1.73 m^2^	3	10	15	11	0	2.87
Not necessary in case of confirmed LN class III, IV, or V	4	11	11	4	6	2.92
At the end of induction therapy:	0	0	7	8	7	4.00
• regardless of response to therapy	0	0	0	10	19	4.66
• in case of only partial response after 6–12 months	7	15	9	3	2	2.39
• in case of no response after 3–4 months	6	16	10	3	0	2.29
In remission prior to discontinuation of maintenance therapy	0	0	2	12	25	4,59

### Concomitant Therapy and Preventive Measures for Lupus Nephritis

There was general agreement that patients should receive HCQ as a concomitant therapy for LN (Table 7). Other important measures included therapy with an angiotensin-converting enzyme (ACE) inhibitor or angiotensin II (ATII) receptor antagonist in cases of arterial hypertension and/or proteinuria, followed by gonadotropin releasing hormone (GnRH) analogs or fertility preservation, prior to therapy with CP, prescription of progestogen (especially in antiphospholipid syndrome), vitamin D substitution and the implementation of vaccinations and infection prophylaxes.

**TABLE 7:** Useful concomitant therapies or preventive measures in patients with lupus nephritis (answers by Likert scale: 1 = very important, 2 = somewhat important, 3 = not very important, 4 = not important at all; with rating average of respondents).

	1 = very important; total number of respondents	2 = somewhat important; total number of respondents	3 = not very important; total number of respondents	4 = not important at all; total number of respondents	Rating average
Hydroxychloroquine	37	5	0	0	1.12
ACE inhibitor or ATII receptor antagonist in case of arterial hypertension	34	7	0	0	1.17
Indication vaccinations (e.g., influenza, pneumococcus)	26	15	0	0	1.34
Sperm or oocyte preservation before CP	16	9	3	0	1.54
Passive use of Low Molecular Weight Heparin (LMWH) (in case of immobility and/or nephrotic syndrome)	21	15	5	0	1.61
Low-dose acetylsalicylic acid (ASA) in case of positive antiphospholipid antibodies	17	21	3	0	1.66
GnRH analogs in post-pubertal patients and CP	19	13	5	1	1.68
Pneumocystis prophylaxis under CP	19	15	6	0	1.68
IgG substitution in case of IgG deficiency after RTX	19	16	6	0	1.68
ACE inhibitor or ATII receptor antagonist in case of proteinuria	7	15	1	0	1.74
Calcium supplementation	20	7	9	1	1.76
Gynecology consult for Post-pubertal patients once yearly with Pap smear	16	17	6	1	1.80
Vitamin D	9	10	8	0	1.96
• fixed dose of 1000 IU/d	10	10	11	1	2.09
• level-adapted (target 30 μ g/l or 75 nmol/l)	5	18	16	2	2.37
Pneumocystis prophylaxis under RTX	13	14	10	1	1.97
Start contraception for patients of childbearing age	8	16	7	0	1.97
• always a progestogen-only contraceptive pill	6	8	6	4	2.33
• progestogen-only contraceptive pill only if antiphospholipid antibodies are positive	15	19	5	1	1.80
Monitoring of CMV viral load in relapses of the underlying disease or before intensification of immunosuppression	6	23	10	1	2.15

## Discussion

This study provides new insights into treatment practices of pediatric subspecialists caring for patients with proliferative LN class III and IV in Germany and Austria. As early diagnosis and prompt treatment of LN can improve long-term renal survival (19), the two working groups (PRO-KIND SLE working group and the GPN SLE working group) have the following common goal: to develop consensus protocols for clinical practice with clearly defined treatment goals including timelines for achieving these goals.

The principle of T2T has been successfully introduced for several rheumatic diseases including juvenile idiopathic arthritis, recently published (10–15). Identifying appropriate therapeutic targets and translation of these targets into clinical practice will lead to improved care for patients and, subsequently, to a better outcome (20). International and German consensus treatment recommendations for LN in children are available (16, 17, 21, 22), but lack a T2T approach.

Overall, the answers to the survey questions reflect a large heterogeneity in the management of juvenile proliferative LN in Germany and Austria, supporting the need to design T2T strategies which should be consented by the relevant subspecialties caring for SLE patients.

Rheumatologists participating in this survey are more likely to involve a nephrologist in the diagnosis and treatment planning of an SLE patient with suspected LN than vice versa, while international recommendations emphasize the inclusion of both disciplines (16, 21). That only 55% consult a rheumatologist might also be partly due to the fact that the majority of respondents are themselves pediatric rheumatologists. Since isolated lupus nephritis is a very rare condition (23–25) and SLE is a multisystem disease, interdisciplinary collaboration is important and worthy of support, to which the joint establishment of a treatment protocol could contribute.

When assessing the extent of LN, protein excretion in 24 h collection urine (>300 mg/m^2^ per 24 h or ≥0.5 g per 24 h) and protein-creatinine ratio (>0.2 g/g creatinine or >20 mg/mmol) in spot urine collection were evaluated as key parameters. Recently, Smith et al. did not find significance of proteinuria in differentiating SLE patients with and without development of LN longitudinally (26). However, proteinuria is generally noted in patients with juvenile proliferative LN (7) and remains an important tool for detecting subclinical renal involvement in SLE (16). In our survey, serum creatinine and eGFR were also assessed as important parameters in evaluating the extent of LN. It is worth mentioning, that these two parameters failed to discriminate between patients with and without LN in the United Kingdom JSLE Cohort Study (26).

The definition of therapeutic targets is obviously a core element of the T2T approach. To date, the literature does not offer a uniform definition of complete remission in LN. The Joint European League Against Rheumatism and European Renal Association-European Dialysis and Transplant Association (EULAR/ERA-EDTA) recommendations define proteinuria as <0.5 g/24 h and normal or near normal (within 10% of normal eGFR if previously abnormal) eGFR as complete response (CR), and this definition has also been adopted by the SHARE Initiative (16, 27). In our survey, respondents also rated the increase of eGFR to >90 ml/min/m^2^ as a very important target for remission, as well as protein excretion in 24 h urine collection < 200 mg/24 h, which is somewhat stricter than the above recommendations. Furthermore, normalization of serum complement C3 is also an important decision tool to define CR to the respondents, while it has been shown to have modest specificity for active LN (28). On the other hand, in a recent British cohort, C3 levels at baseline were a significant predictor for subsequent LN development (26). In summary, targets in the management of LN need to be defined and should be the subject of consensus findings.

When renal involvement is suspected in patients with SLE, kidney biopsy is the widely accepted gold standard (16, 29). In our survey, respondents stipulated that nephrotic-range or rapidly progressing proteinuria were important indicators for kidney biopsy, reflecting the consensus in adult SLE (27, 30–32). The fact that respondents of this survey failed to rate ethnicity as an important parameter in the indication for kidney biopsy, is in discordance with the literature: African Americans, East Asians, and Hispanics with SLE are more likely to develop LN than are SLE patients of European descent (33, 34). This result may reflect the historically lower prevalence of these ethnicities in Germany and Austria and the lack of awareness of this risk factor. Many pediatric nephrologists and rheumatologists continue to follow ACR recommendations when deciding on the necessity of a kidney biopsy (17, 35).

For induction therapy of proliferative LN, our survey suggests that the use of corticosteroids is mandatory, with respondents preferring combined intravenous and oral administration. The majority of participants suggested maintaining PDN treatment for at least one year, but consider discontinuation thereafter, in order to avoid long-term side effects (36). However, there was no consensus on optimal dose and duration of PDN treatment in children in order to balance efficacy and side effects. A recent study conducted by the GPN in children with LN class III or IV, showed corticosteroid toxicity in 42% and growth failure in 78% of children in the first year of treatment (7). Therefore, a consensus for optimal corticosteroid dosing is of utmost importance.

Mycophenolate mofetil was clearly preferred over CP for induction therapy, in addition to corticosteroids in patients with LN class III. By contrast, CP (0.5 g/m^2^/month for 6 months) and MMF were rated equally for induction treatment of LN class IV. This approach is supported by recent registries and cohort studies suggesting the comparability of MMF and CP in induction therapy for proliferative LN in children, although no difference between MMF and CP with respect to treatment-associated side effects was noted with a follow-up of maximum 13 months (7, 37, 38). The use of high-dose intravenous CP (0.5–0.75 g/m^2^ monthly for 6 months) was recommended to be reserved for adult patients with proliferative LN class III/IV showing unfavorable clinical (nephritic urine sediment and impaired renal function with an eGFR between 25 and 80 ml/min/1.73 m^2^), or histologic (crescents or necrosis in >25% of glomeruli) prognostic factors (21). Of note, there was no consensus on the MMF dosage regimen to be used during induction treatment with half each of the participants proposing 2 and 3 g, respectively. The latter was more frequently proposed by pediatric nephrologists and is in line with recent guidelines for treatment of proliferative LN in adults (21).

Most physicians considered an interval of 12 weeks as acceptable to assess treatment response, which is in agreement with a survey of North American pediatric nephrologists and rheumatologists (39). This timeline may be rather optimistic, as recent registry data showed that 25 and 17% of German patients with juvenile LN class III/IV receiving induction treatment with corticosteroids in combination with either MMF of CP showed persistent proteinuria after 3 and 6 months, respectively (7). Again, consensus on timelines for treatment targets need to be better defined. This is important, as it will guide physicians toward switching to second-line treatments.

In case of non-response to induction therapy, most respondents opt for a switch of medication beyond the increase of the corticosteroid dose, depending on previous therapy. This most often included RTX (preferred by 8% among nephrologists vs. 25% among rheumatologists) (39), followed by CP, MMF, and CsA, whereas other measures such as plasmapheresis and immunoadsorption were rarely proposed. This reflects what is currently recommended for adult patients with proliferative LN in case of treatment failure or partial response only, i.e., switching to MMF, a calcineurin inhibitor, intravenous CP or RTX (21, 40–49).

Mycophenolate mofetil was most often recommended for maintenance therapy in LN class III/IV, in conjunction with oral corticosteroids, whereas AZA or CsA were rarely suggested, which is in line with recommendations for adults with proliferative LN (21, 49).

As for concomitant therapies and preventive measures in childhood LN, results of our survey echo the published data, that pediatric SLE patients should all be treated with HCQ (50). As there is evidence in adult SLE patients that ACE inhibitors or ATII receptor antagonist have a protective effect on the kidneys in case of proteinuria (51, 52), its use is recommended in children with LN and proteinuria (16), a view widely shared by respondents of this survey. In addition, respondents of this survey confirm the importance of using inhibitors of the renin-angiotensin system in arterial hypertension, as documented in international recommendations (53).

The question of fertility preservation in therapy with CP is particularly relevant in adolescent patients. Low-dose intravenous CP does not seem to impact ovarian reserve as measured by anti-Mullerian hormone (54) and the SHARE initiative did not include recommendations for fertility preservation (16). Still, the occurrence of premature ovarian failure (POF) and the risk of permanent sterility in young men the with CP exposure is a rare but serious event (55, 56). In addition to the CP dose limitation that appears to minimize the risk of fertility reduction (57), the combined use of GnRH analogs with CP therapy was shown to be associated with a significant reduction of POF among premenopausal women with SLE, suggesting that the addition of GnRH analog can be a strategy to prevent POF among premenopausal women (58). In addition to endorsing this measure, participants of the survey also consider sperm or oocyte preservation before CP to be useful which, of course, must be discussed individually with each patient (59).

It was shown that low-dose ASA may be beneficial in the primary prophylaxis of cardiovascular (CV) events in SLE patients (60, 61). Considering the general increased risk for a CV event in SLE patients (62) and especially with positive antiphospholipid antibodies, a low-dose acetylsalicylic acid (ASA) therapy in case of positive antiphospholipid antibodies can be considered useful, as suggested by our respondents and also in the literature (50).

The limitations of our study are low participation/response rates (which is not unusual for an online survey distributed *via* mail) and which may be related to the treatment of LN being primarily in highly specialized centers. However, LN in children and adolescents is a rare condition. Therefore, the number of rheumatologists and nephrologists treating children with LN is also low and likely only those felt consequently addressed to answer the survey. In addition, there is a possible selection bias in only addressing members of the mailing lists of GKJR and GPN. We realize that the definitions of treatment response and failure need to be more clearly delineated. Finally, the role of adherence and therapeutic drug monitoring to optimize treatment with MMF, and the use of “multitarget therapy” (i.e., MMF in combination with a calcineurin inhibitor) for induction of LN were not included in this survey, as these measures have only recently gained attention. The same accounts for new therapeutic options, such as belimumab, which has been approved as an add-on therapy for adult SLE patients with LN in Germany since 2021. In this 2017 survey, belimumab was not yet considered as a treatment option, but which may gain importance in childhood LN therapy.

Several additional aspects should be discussed when treating children and adolescents with LN, such as treatment adherence (possibly promoting intravenous drug administration), the issue of growth (corticosteroid dose limitation), fertility, necessitating CP dose limitation, as well as the psychosocial aspects, such as schooling and socialization with peers. In a study conducted by the GPN in children with LN class III or IV, 80% of patients had drug-related complications in the first year of treatment, including glucocorticoid toxicity in 42% of children and growth retardation in 78% (7).

In conclusion, our survey reveals that the majority of German and Austrian pediatric rheumatologists and nephrologists would use corticosteroids, most often in combination with either MMF or CP for induction treatment of juvenile proliferative LN. Minimization of steroid-exposure remains a major challenge in these children and adolescents, asking for well-designed clinical trials to define the optimal dosage and duration of corticosteroid treatment. The considerable heterogeneity of responses highlights the need for a treat-to-target protocol (T2T) between pediatric rheumatologists and nephrologists. This goal is to be achieved, among other measures, through interdisciplinary cooperation in consensus conferences followed by either controlled or register studies, in which the value of the T2T protocols is tested.

## Data Availability Statement

The original contributions presented in the study are included in the article/Supplementary Material, further inquiries can be directed to the corresponding author.

## Author Contributions

CS, FS, CMH, CH, and KT designed the survey. KM, JB, UN, AH, TK, NW, BT, LW, and DH revised the survey design. CS, KT, and KV were involved in interpretation of the data and analyzed the data. KV drafted the manuscript. KT, CMH, BT, FS, and DH substantively revised the manuscript. All authors critically revised the manuscript and approved the final draft.

## Conflict of Interest

The authors declare that the research was conducted in the absence of any commercial or financial relationships that could be construed as a potential conflict of interest.

## Publisher’s Note

All claims expressed in this article are solely those of the authors and do not necessarily represent those of their affiliated organizations, or those of the publisher, the editors and the reviewers. Any product that may be evaluated in this article, or claim that may be made by its manufacturer, is not guaranteed or endorsed by the publisher.

## Abbreviations

ACE: angiotensin-converting enzyme
ACR: American College of Rheumatology
ASA: acetylsalicylic acid
ATII: angiotensin II
AZA: azathioprine
BILAG: British Isles Lupus Assessment Group index
BSA: Body Surface Area
CARRA: Childhood Arthritis and Rheumatology Research Alliance
C-HAQ: Childhood Health Assessment Questionnaire
CP: cyclophosphamide
CR: complete response
CsA: cyclosporin A
CTP: consensus treatment plan
CV: cardiovascular
ECLAM: European Consensus Lupus Activity Measurement
eGFR: estimated glomerular filtration rate
ERA-EDTA: European Renal Association-European Dialysis and Transplant Association
EULAR: Joint European League Against Rheumatism
GKJR: Society for Pediatric and Adolescent Rheumatology
GnRH: gonadotropin releasing hormone
GPN: German Society for Pediatric Nephrology
HCQ: hydroxychloroquine
IVIG: intravenous immunoglobulin
LMWH: low molecular weight heparin
LN: lupus nephritis
MMF: mycophenolate mofetil
MP: methylprednisolone
NSI: non-steroidal immunosuppression
PDN: prednisolone
POF: premature ovarian failure
PRO-KIND: Projekte zur Klassifikation Überwachung und Therapie in der Kinderrheumatologie/Projects on classification monitoring and therapy in pediatric rheumatology
RBC casts: Red Blood Cell casts
RTX: rituximab
SHARE: Single Hub and Access point for pediatric Rheumatology in Europe
SLE: systemic lupus erythematosus
SLEDAI-2K: Systemic Lupus Erythematosus Disease Activity Index 2000
SMILEY: Simple Measure of Impact of Lupus Erythematosus in Youngsters
T2T: treat-to-target
TAC: tacrolimus.
