# Supplementary material for: Working Towards a Treat-to-Target Protocol in Juvenile Proliferative Lupus Nephritis – A Survey of Pediatric Rheumatologists and Nephrologists in Germany and Austria
Source: Front Pediatr. 2022 Apr 22;10:851998. doi: 10.3389/fped.2022.851998 (PMC9072733; doi:10.3389/fped.2022.851998)
Supplement: Supplementary file 1 [file Data_Sheet_1.pdf]

Appendix

**Full survey**

**I. Case record**

History and physical status:

A 15-year-old previously healthy girl presents in your clinic with fatigue, subfebrile temperatures, weight loss, and a sun-induced exanthema of the face, which occurred five months ago. The patient complains of bilateral pain in several finger joints and of pain in knees and ankles.

On physical examination, one can observe impaired general condition, malar rash, oral ulcers, diffuse finger swelling but no arthritis in the other remaining joints.

General laboratory parameters:

Leukocytes 2.600/µl, neutrophils 1.400/µl, lymphocytes 800/µl, Hb 10g/dl, platelets 90.000/µl, ESR 65/120mm, CRP 1.0 mg/dl, IgG 28g/L, C3 and C4 <50% of age norm, creatinine 1.1 mg/dl (97 mol/l), ferritin slightly decreased. Transaminases, uric acid and lactate dehydrogenase are within normal range. Urine sediment with 10 erythrocytes/field, urine dip-stick test for protein 2-fold positive.

Autoantibodies:

ANA 1:5120, anti-dsDNA strongly positive. Anti-U1RNP and antiphospholipid antibodies negative.

Diagnosis:

You strongly suspect the diagnosis of systemic lupus erythematosus (SLE) and 7/11 of the ACR classification criteria are met.

**II. Diagnostics**

1. Which consultants would you involve in diagnosis and treatment planning (multiple answers possible)?
   - Pediatric nephrologist
   - Pediatric rheumatologist
   - hematologist/ hemostaseologist
   - Pediatric cardiologist
   - Pediatric pulmonologist
   - Pediatric endocrinologist
   - ophthalmologist (with fundus)
   - neuropediatrician
   - psychologist/ psychiatrist
   - dermatologist
   - gynecologist
   - other (please name):__________________

2. Please rate the following laboratory parameters in terms of their importance in assessing the extent of lupus nephritis. Answers by Likert scale: 1=very important, 2=somewhat important, 3= not very important, 4=not important at all
   - Urine dip-stick: Protein >2-fold positive
   - Spontaneous urine: protein-creatinine ratio >0.2 g/g creatinine
   - 24h collection urine: >300 mg/m² and 24h or ≥ 0.5g in 24h
   - Serum creatinine
   - Schwartz formula: estimated GFR (eGFR)
   - Cystatin C: estimated GFR (eGFR)

- creatinine clearance/BSA in 24h collection urine

**III. Indication for kidney biopsy in SLE patients**

3. Which urine findings would you consider as an indication for kidney biopsy in SLE patients?
    - I initially order a kidney biopsy in every patient, regardless of urine and kidney parameters.
    - I perform a biopsy only in case of relevant pathological urine and kidney parameters
    - I perform a biopsy despite relevant pathological urine and kidney parameters only if there is an insufficient response to therapy after 3-6 months

4. For which urine findings would you consider as an indication for kidney biopsy (multiple answers possible)?
    - isolated pathologic urine status (e.g., >5 erythrocytes/high power field and/or detection of RBC casts) without proteinuria
    - isolated mild-moderate proteinuria (≤1 g/m2 and 24h or protein-creatinine ratio 0.2-2 g/g creatinine) with normal urine status
    - pathological urine status (see above) and mild-moderate proteinuria (see above)
    - nephrotic-range proteinuria (>1 g/m² and 24h or protein-creatinine ratio >2 g/g creatinine) independent of urine status
    - rapidly progressive proteinuria
    - other:_________________

5. What other parameters are relevant to you in deciding whether to have a kidney biopsy performed? Answers as Likert scale: 1=very important, 2=somewhat important, 3= not very important, 4=not important at all
    - strongly increased anti-dsDNA
    - strongly decreased C3
    - strongly decreased C4
    - the combination of high auto-antibodies (dsDNA and/or nucleosomes) plus decreased complement levels (C3 and/or C4) is crucial for me
    - elevated serum creatinine levels
    - eGFR <90ml/min/1.73m²
    - eGFR <60ml/min/1.73m²
    - elevated blood pressure (>95th percentile)
    - Patient ethnicity (African-American, Hispanic, Asian)

6. What other parameters would be relevant to you in the decision process to have a kidney biopsy performed in the event of a suspected flare? Answers by Likert scale: 1=very important, 2=somewhat important, 3=not very important, 4=not important at all)
    - Increase of anti-dsDNA antibodies
    - Increase in proteinuria: e.g. nephrotic-range proteinuria with previously mild-moderate proteinuria
    - new onset of mild-moderate proteinuria
    - newly appeared RBC casts or macroscopic hematuria
    - severely decreased C3 and/or C4
    - the combination of high autoantibodies (dsDNA and/or nucleosomes) plus decreased complement levels (C3 and/or C4)

- elevated serum creatinine levels
- eGFR <90ml/min/1.73m² with prior eGFR > 90ml/min/1.73m²
- eGFR <60ml/min/1.73m² if previously eGFR > 90ml/min/1.73m²
- elevated blood pressure (> 95th percentile)

**III. Activity assessment of SLE**

7. Which of these validated tools do you use to assess SLE (multiple answers possible)?
   - Systemic Lupus Erythematosus Disease Activity Index 2000 (SLEDAI-2K)
   - British Isles Lupus Assessment Group (BILAG) index
   - European Consensus Lupus Activity Measurement (ECLAM)
   - Simple Measure of Impact of Lupus Erythematosus in Youngsters (SMILEY)
   - Pediatric Quality of Life Inventory Multidimensional Fatigue Scale (PedsQL)
   - Physician global assessment
   - Parent/Patient Global Assessment
   - Childhood Health Assessment Questionnaire (C-HAQ)
   - Child Health Questionnaire (CHQ)
   - other:_________________

**IV. Therapy for proliferative lupus nephritis class III or IV**

Three possible corticosteroid induction therapies are described in details in the following table, and are referred to in the case vignette.
Table: adapted from Mina R, Consensus treatment plans for induction therapy of newly diagnosed proliferative lupus nephritis in juvenile systemic lupus erythematosus. Arthritis Care Res (Hoboken) 2012;64(3):375-83 and the consensus paper of the Association for Pediatric Nephrology (GPN), Therapy Recommendation for Lupus Nephritis in Children and Adolescents 2007

| | **Prednisolone/methylprednisolone (PDN/MP) therapy in the first 6 months** |
|---|---|
| **Mainly intravenous (i.v.)** | MP i.v. 15-30 mg/kg (max 1g) or 300-500 mg/m² for 3 days,<br>then i.v. MP pulse therapy initially 1x/week, then 1x/month<br>+<br>start PDN per os (p.o.) 0.5mg/kg → reduction → target PDN 6-10 mg/m²/48h or 0.2mg/kg (up to max 10mg/d) p.o. |
| **Mainly p.o.** | MP once i.v. 15-30mg/kg (max 1g) or 300-500 mg/m² for 3 days<br>+<br>start PDN p.o. 2mg/kg or 60mg/m² for 6 weeks → reduction → target PDN 6-10 mg/m²/48h or 0.5mg/kg (max 20mg/d) |
| **Combined i.v. + p.o.** | MP i.v. 15-30mg/kg (max 1g) or 300-500 mg/m² for 3 days,<br>further optional i.v. MP pulse therapy (max 1x/month)<br>+<br>p.o. PDN start 1(-1.5)mg/kg → reduction by 10% every (1-)2 weeks → target PDN 6-10 mg/m²/48h or 0.2mg/kg (up to max. 15mg/d) |

**IV. case 1: lupus nephritis class III**

The 15-year-old female patient presenting with symptoms of SLE described above shows the following renal parameters: Histology with LN WHO class III, proteinuria 500mg/m² and protein-/creatinine ratio

0.8 g/g creatinine, erythrocyte cylinder: 10/high power field, eGFR: 110ml/min/1.73$m^2$, blood pressure 75th percentile

8. Which induction therapy would you choose?
Please tick first which form of corticosteroid therapy you prefer and then if and which additional immunosuppression you would use (multiple answers are possible for the latter).
   - prednisolone/methylprednisolone (see above for definition)
     a. mainly i.v.
     b. mainly p.o.
     c. combined i.v. + p.o.
   - no additional immunosuppression
   - PDN/MP plus additional immunosuppression
     a. CP iv
     b. MMF
     c. CSA
     d. AZA
     e. RTX
     f. other:_________________

9. Which maintenance therapy would you choose?
   - discontinue PDN p.o. when nephritis is in complete remission
   - prednisolone
     a. fixed low-dose p.o. PDN dose for another year
        - 6-10 mg/$m^2$/48h
        - 2.5-3.75mg/d or <0.15mg/kg
        - 5-7.5mg/d or 0.15-0.2mg/kg
        - >7.5mg/d or >0.2 mg/kg
   - other immunosuppression
     a. CP
     b. MMF
     c. AZA
     d. CSA
     e. RTX
     f. other: ________________

10. How would you react in case of non-response to induction therapy (see also V)? If you would combine immunosuppressants, please indicate in the respective free text field.
   - Isolated increase of p.o. PDN and/or i.v. MP and keep current immunosuppressive baseline medication.
   - Combination of p.o. PDN and/or i.v. MP plus change of basic immunosuppressive medication to:

   - in case of primary treatment with MMF
     a. CP i.v.
        - 0.5g/m2/month for 6 months
        - 0.75-1g/m2/month for 6 months
        - EUROLUPUS scheme: 6 x 500mg every 14 days

- 1-3 CP doses since early combination with other immunosuppression (see below)
- other dosing_________________

b. CSA
c. RTX

- in case of primary treatment with CP
    a. MMF
    b. CSA
    c. RTX

- in case of primary treatment with CSA
    a. MMF
    b. RTX
    c. CP i.v.

- AZA
- plasmapheresis
- immunoadsorption
- other:________________________________________
- I choose a combination of multiple immunosuppressants (free text entry): ________________________________________________________________

**IV. case 2: lupus nephritis class IV (with additional prognostically unfavorable risk factors)**

The 15-year-old female patient presenting with symptoms of SLE described above shows the following renal parameters: Histology with LN WHO class IV and 50% crescent formation, proteinuria 1.5 g/m$^2$ and protein-creatinine ratio 2.1 g/g creatinine, erythrocyte cylinder 10/high power field (positive), eGFR 72ml/min/1.73m$^2$, blood pressure 97th percentile.

11. Which induction therapy would you choose? Please tick first which form of corticosteroid therapy you prefer and then whether and which additional immunosuppression you would use (multiple answers are possible).
    - prednisolone/methylprednisolone (see above for definition)
        a. mainly i.v.
        b. mainly p.o.
        c. combined i.v. + p.o.
    - no additional immunosuppression
    - PDN/MP plus additional immunosuppression
        a. CP i.v.
            i. 0.5g/m$^2$/month for 6 months
            ii. 0.75-1g/m$^2$/month for 6 months
            iii. EUROLUPUS scheme: 6 x 500mg every 14 days
            iv. only 1-3 CP doses, due to early combination with other immunosuppression (see below)
            v. other dosage_________________
    - MMF 1000-1200mg/m$^2$, max 3 g/d
    - MMF 1000-1200mg/m$^2$, max 2 g/d

- CSA
- AZA
- RTX
- plasmapheresis
- immunoadsorption
- other:_________________
- I choose a combination of multiple immunosuppressants (free text entry): _________________________________________________________

12. Which maintenance therapy (after reaching inactive disease within six months) would you choose?
    - prednisolone
        a. low-dose PDN p.o. dose (at least for another year)
            i. >7.5mg/m²/d or 0.2 mg/kg
            ii. 5 - 7.5mg/m²/d or 0.15-0.2 mg/kg
            iii. 2.5 - 3.75mg/m²/d or <0.15 mg/kg
            iv. 6-10 mg/m²/48h
        b. Discontinuation of PDN p.o. at complete remission of nephritis
    - further immunosuppression
        a. CP 0.5-1g/m² once every 3 months for 2 years
        b. MMF
            i. 1000-1200mg/m² (max 2g/d)
            ii. 600-900 mg/m²
        c. CSA
        d. AZA
        e. RTX
            i. preemptive 2nd RTX cycle after 3-6 months
            ii. 2nd RTX cycle after B-cell repopulation
            iii. 2nd RTX cycle only in case of clinical recurrence

13. Which procedure would you choose in case of non-response to induction therapy (see also V)? If you would combine immunosuppressants, please indicate in the respective free text field.
    - Isolated increase of p.o. PDN and/or i.v. MP and keep current immunosuppressive baseline medication.
    - Isolated increase of p.o. PDN and/or i.v. MP and maintenance of current immunosuppressive basic medication
    - Combination of p.o. PDN and/or i.v. MP plus change in baseline immunosuppressive medication to

        a. for primary treatment with MMF
            i. CP i.v.
                - 0.5g/m²/month for 6 months
                - 0.75-1g/m²/month for 6 months
                - EUROLUPUS scheme: 6 x 500mg every 14 days
                - only 1-3 CY doses since early combination with other immunosuppression (see below)
                - other dosing_________________
            ii. CSA

iii. RTX

b. in case of primary treatment with CP
   i. MMF
   ii. CSA
   iii. RTX

c. in case of primary treatment with CSA
   i. MMF
   ii. RTX
   iii. CP i.v.
      - $0.5g/m^2$/month for 6 months.
      - $0.75\text{-}1g/m^2$/month for 6 months.
      - only 1-3 CP doses since early combination with other immunosuppression (see below)
      - other dosing_________________

- AZA
- plasmapheresis
- immunoadsorption
- other:________________________________________
- I choose a combination of multiple immunosuppressants (free text entry): ______________________________________________________________

## V. Definition of response to therapy in lupus nephritis

14. What criteria do you use to assess remission? Please rate the following parameters in terms of their importance.
    Answers by Likert scale: 1=very important, 2=somewhat important, 3=not very important, 4=not important at all
    - Urine sediment (erythrocytes 5/high power field, no RBC casts detectable)
    - Protein-creatinine ratio <0.2 g/g creatinine or protein excretion <200mg/24h in 24h urine collection
    - eGFR >90ml/min/$1.73m^2$
    - normalization of serum complement C3
    - SLEDAI score <2

15. From my point of view, the following time interval until the onset of therapy is allowable before further action is needed.

    For case 1 (class III lupus nephritis and proteinuria 0.8 g/g Krea).
    Target: proteinuria < 0.2 g/g Krea and eGFR > 90 ml/min/$1.73m^2$
    - 2 weeks
    - 4 weeks
    - 8-12 weeks
    - 24 weeks
    - 36 weeks
    - 52 weeks

For case 2 (class IV lupus nephritis and proteinuria 2.1 g/g Krea).
Target: proteinuria < 0.2 g/g Krea and eGFR > 90 ml/min/1.73m²

- 2 weeks
- 4 weeks
- 8-12 weeks
- 24 weeks
- 36 weeks
- 52 weeks

**Maintenance therapy**

16. How long do you perform immunosuppressive maintenance therapy of lupus nephritis in case of good response?
    - 1 year
    - 3 years
    - ≥3 years

**Control kidney biopsy**

17. In what situation would you consider a control kidney biopsy for lupus nephritis?
    Answers by Likert scale: 1=fully agree, 2=tend to agree, 3=partly / partly, 4= tend to disagree, 5=do not agree at all

    - not necessary in case of once confirmed class III, IV or V lupus nephritis

    - in case of suspected recurrence of nephritis
    - at the end of induction therapy:
        a. regardless of response to therapy
        b. in case of only partial response after 6-12 months
        c. in case of no response after 3-4 months
    - in maintenance therapy if proteinuria persists >1 year
    - in remission prior to discontinuation of maintenance therapy
    - persistent eGFR <90 ml/min/1.73m²

**VI. Concomitant Therapy in SLE or lupus nephritis**

18. Which of the following supportive therapies or preventive measures would you consider useful in patients with lupus nephritis?
    Answers by Likert scale: 1=very important, 2=somewhat important, 3=not very important, 4=not important at all
    - hydroxychloroquine
    - ACE inhibitor or ATII receptor antagonist in arterial hypertension
    - ACE inhibitor or ATII receptor antagonist in proteinuria
    - vitamin D
        a. fixed dose of 1000IE/d
        b. level-adapted (target 30µg/l or 75 nmol/l)
    - calcium supplementation
    - GnRH analogues in postpubertal patients and CP

- sperm or oocyte preservation before CP
- start contraception for patients of childbearing age
    a. always a progestogen-only contraceptive pill
    b. progestogen-only contraceptive pill only if antiphospholipid antibodies are positive
- gynecology consult for postpubertal patients 1x per year with Pap smear
- low-dose acetylsalicylic acid (ASA) in case of positive antiphospholipid antibodies
- passive use of low molecular Heparin (in case of immobility and/or nephrotic syndrome)
- pneumocystis prophylaxis under CP
- pneumocystis prophylaxis under RTX
- indication vaccinations (e.g. influenza, pneumococcus)
- monitoring of CMV viral load in relapses of the underlying disease or before intensification of immunosuppression
- IgG substitution in case of IgG deficiency after RTX
